# Radioimmunotherapy of micrometastases in lung with vascular targeted^213^Bi

**DOI:** 10.1038/sj.bjc.6690337

**Published:** 1999-04

**Authors:** S J Kennel, R Boll, M Stabin, H M Schuller, S Mirzadeh

**Affiliations:** Life Sciences Division, Oak Ridge National Laboratory, PO Box 2008, MS-6101, Oak Ridge, TN 37831-6101, USA; Oak Ridge Institute for Science and Education, Medical Sciences Division, PO Box 117, Oak Ridge, TN 37831-0117, USA; University of Tennessee, College of Veterinary Medicine, Experimental Oncology Laboratory, PO Box 1071, Knoxville, TN 37901, USA

**Keywords:** vascular targeting, α-particle emitter, ^213^Bi, therapy

## Abstract

A model system has been used to test the efficacy of vascular targeting of α-particle emitter^213^Bi for therapy of small, ‘artificial’ metastases in mouse lung. Specific monoclonal antibody (mAb) 201B was used to deliver greater than 30% of the injected dose to lung where tumours had developed due to intravenous injection of cells. Specific^213^Bi-mAb 201B treatment of BALB/c mammary carcinoma EMT-6 tumours in lung resulted in a dose-dependent destruction of tumours and an extended lifespan of treated animals relative to controls. Significant reduction of lung tumour burden was noted in animals treated with 0.93 MBq injected dose or as little as 14 Gy absorbed dose to the lung. Animals treated with higher doses (2.6–6.7 MBq) had nearly complete cure of lung tumours but eventually died of lung fibrosis induced by the treatment. Four other tumour cell types were studied: murine Line 1 lung carcinomas in syngeneic BALB/c mice, rat IC-12 tracheal carcinoma growing in severe combined immune deficient (SCID) mice, and two human tumours – epidermoid carcinoma A431 and lung carcinoma A549 – growing in SCID mice. In all cases, the number of lung tumour colonies was reduced in animals treated with specific, labelled mAb relative to those in animals treated with control^213^Bi MAb or EDTA complexed^213^Bi. Tumours treated in immunodeficient SCID mice were partially destroyed or at least retarded in growth, but ultimately regrew and proved fatal, indicating that an intact immune function is necessary for complete cure. The data show that the short-lived α-particle emitter^213^Bi can be effectively targeted to lung blood vessels and that tumour cells growing in the lung are killed. The mechanism may involve direct killing of tumour cells from α-particle irradiation, killing through destruction of blood supply to the tumour, or a combination of the two. © 1999 Cancer Research Campaign


*British Journal of Cancer* (1999) **80**(1/2), 175–184
© 1999 Cancer Research Campaign
Article no. bjoc.1998.0337

### Radioimmunotherapy of micrometastases in lung with vascular targeted $^{213}$Bi

**SJ Kennel[1], R Boll[1], M Stabin[2], HM Schuller[3] and S Mirzadeh[1]**

[1]Life Sciences Division, Oak Ridge National Laboratory, PO Box 2008, MS-6101, Oak Ridge, TN 37831-6101, USA; [2]Oak Ridge Institute for Science and Education, Medical Sciences Division, PO Box 117, Oak Ridge, TN 37831-0117, USA; [3]University of Tennessee, College of Veterinary Medicine, Experimental Oncology Laboratory, PO Box 1071, Knoxville, TN 37901, USA

**Summary** A model system has been used to test the efficacy of vascular targeting of α-particle emitter $^{213}$Bi for therapy of small, 'artificial' metastases in mouse lung. Specific monoclonal antibody (mAb) 201B was used to deliver greater than 30% of the injected dose to lung where tumours had developed due to intravenous injection of cells. Specific $^{213}$Bi-mAb 201B treatment of BALB/c mammary carcinoma EMT-6 tumours in lung resulted in a dose-dependent destruction of tumours and an extended lifespan of treated animals relative to controls. Significant reduction of lung tumour burden was noted in animals treated with 0.93 MBq injected dose or as little as 14 Gy absorbed dose to the lung. Animals treated with higher doses (2.6–6.7 MBq) had nearly complete cure of lung tumours but eventually died of lung fibrosis induced by the treatment. Four other tumour cell types were studied: murine Line 1 lung carcinomas in syngeneic BALB/c mice, rat IC-12 tracheal carcinoma growing in severe combined immune deficient (SCID) mice, and two human tumours – epidermoid carcinoma A431 and lung carcinoma A549 – growing in SCID mice. In all cases, the number of lung tumour colonies was reduced in animals treated with specific, labelled mAb relative to those in animals treated with control $^{213}$Bi MAb or EDTA complexed $^{213}$Bi. Tumours treated in immunodeficient SCID mice were partially destroyed or at least retarded in growth, but ultimately regrew and proved fatal, indicating that an intact immune function is necessary for complete cure. The data show that the short-lived α-particle emitter $^{213}$Bi can be effectively targeted to lung blood vessels and that tumour cells growing in the lung are killed. The mechanism may involve direct killing of tumour cells from α-particle irradiation, killing through destruction of blood supply to the tumour, or a combination of the two.

**Keywords:** vascular targeting; α-particle emitter; $^{213}$Bi; therapy

Therapy of solid tumours using targeting agents such as tumour cell-directed monoclonal antibodies (mAbs) has proven to be difficult due to the relatively low fraction of the total dose delivered specifically to the tumour. It has been postulated for some time (Denekamp, 1984) that the tumour blood vessels are more accessible targets for directed therapy. Recent work has shown that agents which interfere with new blood vessel formation (angiogenesis) are capable of inhibiting tumour growth in experimental animals (Kim et al, 1993; O'Reilly et al, 1995, 1997; Borgström et al, 1996). The drawback of this approach is that once treatment is stopped, the tumours grow rapidly and eventually prove fatal. Another approach utilized immunotoxins specific for tumour blood vessels. In a model system, an immunotoxin, targeted to major histocompatability complex components induced in blood vessels through genetically altered tumour cells, proved to be therapeutic (Burrows and Thorpe, 1994) and infarction and cure of tumours was observed.

For therapy of small metastases, it is desirable to destroy both the tumour blood vessels and the tumour cells. In this work, short-range, high linear energy transfer (LET) radiation ($^{213}$Bi) was targeted to blood vessels feeding tumours in the lung. The mAb used, mAb 201B, binds to murine thrombomodulin (TM). TM is expressed selectively and in large amounts on the lumenal surfaces of capillaries and small blood vessels in the lung. mAb 201B injected intravenously (i.v.) localizes quickly and in high concentration in murine lung (Kennel et al, 1990). This system was used previously to target the β-particle emitter $^{131}$I to lungs bearing tumours, resulting in a marginal therapeutic effect (Blumenthal et al, 1992). The localization characteristics of mAb 201B allow targeting of short half-lived isotopes such as the α-particle emitter $^{213}$Bi ($t^1/_2$ = 45 min), with delivery of a large fraction of the absorbed dose to the lung as a target (Kennel and Mirzadeh, 1997). The model of experimental lung tumour colonies has been used to test the efficiency of vascular-targeted $^{213}$Bi for radioimmunotherapy. In this system, tumour cells injected i.v. lodge in the lung and form colonies. mAb 201B is then used to deliver $^{213}$Bi to all the lung vessels, including those which feed the tumour colonies. The tissue range of the $^{213}$Bi α-particle is 60–100 μ and thus should produce damage not only to lung vessels, but also to the tumour cells adjacent to the vessels. A preliminary paper has shown that high-dose therapy resulted in tumour cures, but that collateral lung damage occurred (Kennel and Mirzadeh, 1998). Dose–response experiments and radioimmunotherapy with different tumour types were necessary to assess the limits of effectiveness of this approach. We show herein that lung tumour colonies can be significantly reduced by this treatment in five different tumour types with relatively low doses of radioisotope and that tumours which are immunogenic in the host can be cured completely.

*Received 31 October 1997*
*Revised 13 May 1998*
*Accepted 21 May 1998*

*Correspondence to:* SJ Kennel

### MATERIALS AND METHODS

### Cells and animals

EMT-6 cells were derived from a BALB/c mammary carcinoma (Rockwell et al, 1972). They were cultured in Dulbecco's Modified Eagle Medium (DMEM) containing 10% fetal bovine serum (FBS) and were used at passage 8–12. Tumour-derived BALB/c Line 1 lung adenocarcinoma cells (Yuhas et al, 1975) designated 498 were developed at ORNL. The cells were cultured in DMEM with 10% FBS. Cells were used at passage 12–22. Both cells lines were tested repeatedly for mycoplasma contamination using a DNA probe (Gen-Probe, Inc., San Diego, CA, USA).

IC-12 rat tracheal carcinoma was derived from an F344 rat undifferentiated carcinoma induced in vivo in a trachea by exposure to DMBA (Terzaghi-Howe, 1987). It was cultured in B1 modified Ham's F12 medium and used at passage 33.

A431 squamous cell carcinoma and A549 lung adenocarcinoma (Giard et al, 1973) were derived from human tumours. The cell lines were obtained from the American Type Culture Collection. A431, cultured in McCoy's 5A medium with 10% FBS, and A549, cultured in F12K medium with 15% FBS, were used at passages 34 and 52 respectively.

BALB/c and ICR severe combined immune deficient (SCID) mice were bred in the ORNL barrier facility and maintained in a specific pathogen-free (SPF) environment in the AAALAC approved facility. Experiments were completed under the institutional animal care and use committee approved protocol 0138.

### mAb and radioisotope mAb 201B to mouse TM, a rat $IgG_{2a}$, and control rat mAb 14 have been described previously (Kennel et al, 1988). mAb 14 was derived from a rat–mouse hybridoma. It has no known binding specificity either in animals or for any tissue culture cells tested. Purified mAb was derivatized with cyclohexyl diethylenetriaminepentaacetic acid (CHXb-DTPA; Brechbiel and Gansow, 1992), kindly provided by Dr Brechbiel (NIH, Bethesda, MD, USA), at an average of 1–2

**Table 1** Tissue distribution and dosimetry of $^{213}$Bi–mAb[a]

| | 1 h | 2 h | 3 h | 6 h | α | β | Cross dose to lung |
|---|---|---|---|---|---|---|---|
| | mAb 201 (specific) % ID per g | | | | Total dose delivered Gy/MBq | | |
| Blood | 1.85 ± 0.07 | 0.73 ± 0.1 | ~0.4 | – | – | – | – |
| Muscle | 0.72 ± 0.01 | 0.65 ± 0.13 | 0.46 ± 0.1 | – | – | – | 0.0012 |
| Liver | 14.0 ± 0.56 | 16.2 ± 1.9 | 16.5 ± 3.8 | 14.8 ± 0.9 | 0.85 | 0.044 | 0.0047 |
| Spleen | 14.8 ± 1.5 | 20.8 ± 1.8 | 19.1 ± 0.4 | 24.6 ± 2.3 | 0.63 | – | – |
| Kidney | 34 ± 0.8 | 36.7 ± 3.2 | 18.1 ± 1.0 | 18.8 ± 1.5 | 1.4 | 0.055 | < 0.001 |
| Lung | 237 ± 24 | 204 ± 19.5 | 235 ± 22 | 210 ± 13 | 15.5 | 0.30 | – |
| | mAb 14 (control) % ID per g | | | | Total dose delivered by Gy/MBq | | |
| Blood | 16.3 ± 3.5 | 16.4 ± 1.1 | 15.3 ± 1.4 | – | – | – | – |
| Muscle | 0.87 ± 0.02 | 0.88 ± 0.01 | 0.52 ± 0.01 | – | – | – | 0.0010 |
| Liver | 40.3 ± 0.7 | 41 ± 4.2 | 36.1 ± 1.1 | 33.6 ± 2.8 | 2.26 | 0.012 | 0.0012 |
| Spleen | 7.95 ± 0.4 | 7.2 ± 0.7 | 6.9 ± 0.2 | 6.0 ± 1.8 | 0.40 | – | – |
| Kidney | 19.1 ± 0.5 | 19.5 ± 0.2 | 12.2 ± 0.8 | 12.0 ± 0.14 | 0.83 | 0.003 | < 0.001 |
| Lung | 7.1 ± 0.2 | 8.4 ± 1.9 | 5.4 ± 0.5 | 4.5 ± 0.1 | 0.50 | 0.010 | – |

[a]For each time point, BALB/c mice, injected i.v. 6 days previously with $2 \times 10^4$ EMT-6 tumour cells, were injected with 4.1 MBq $^{213}$Bi on 14 μg of either mAb 201B or control mAb 14. Chelation efficiency ranged from 86 to 91% and the preparations were injected after dilution from the chelation reaction without further purification.

**Table 2** Dose–response for treatment of EMT-6 tumours in BALB/c mice

| Treatment[a] | | | Lung tumours | |
|---|---|---|---|---|
| mAb | μg | $^{213}$Bi (mBq) | Animals with tumours number treated | Tumours per lung[b] |
| 201B | 33 | 0 | 8/8 | 78.3 ± 10 |
| 201B | 4 | 0.925 | 5/7 | 12 ± 5 |
| 201B | 8 | 2.6 | 1/7 | 13 |
| 201B | 4/11/7 | 1.0/1.0/1.0 | 7/7 | 2 (> 200)<br>5 (14 ± 8) |
| 201B | 16 | 3.33 | 0/8 | – |
| 201B | 33 | 6.7 | 0/7 | – |
| 14 | 20 | 4.3 | 3/7 | 30 ± 8 |
| 14 | 40 | 7.0 | 0/7 | – |

[a]BALB/c Bd ♀ mice were injected i.v. with $4 \times 10^4$ EMT-6 (passage 8) cells. Animals were treated 5 days after cell injection with i.v. injection of the labelled mAb in 200 μl MES. The fractionated doses were given at 3-day intervals. [b]Average number ± s.d. of tumours counted in histology sections from lungs of tumour-positive animals (Experiment 25 October 1996).

© Cancer Research Campaign 1999

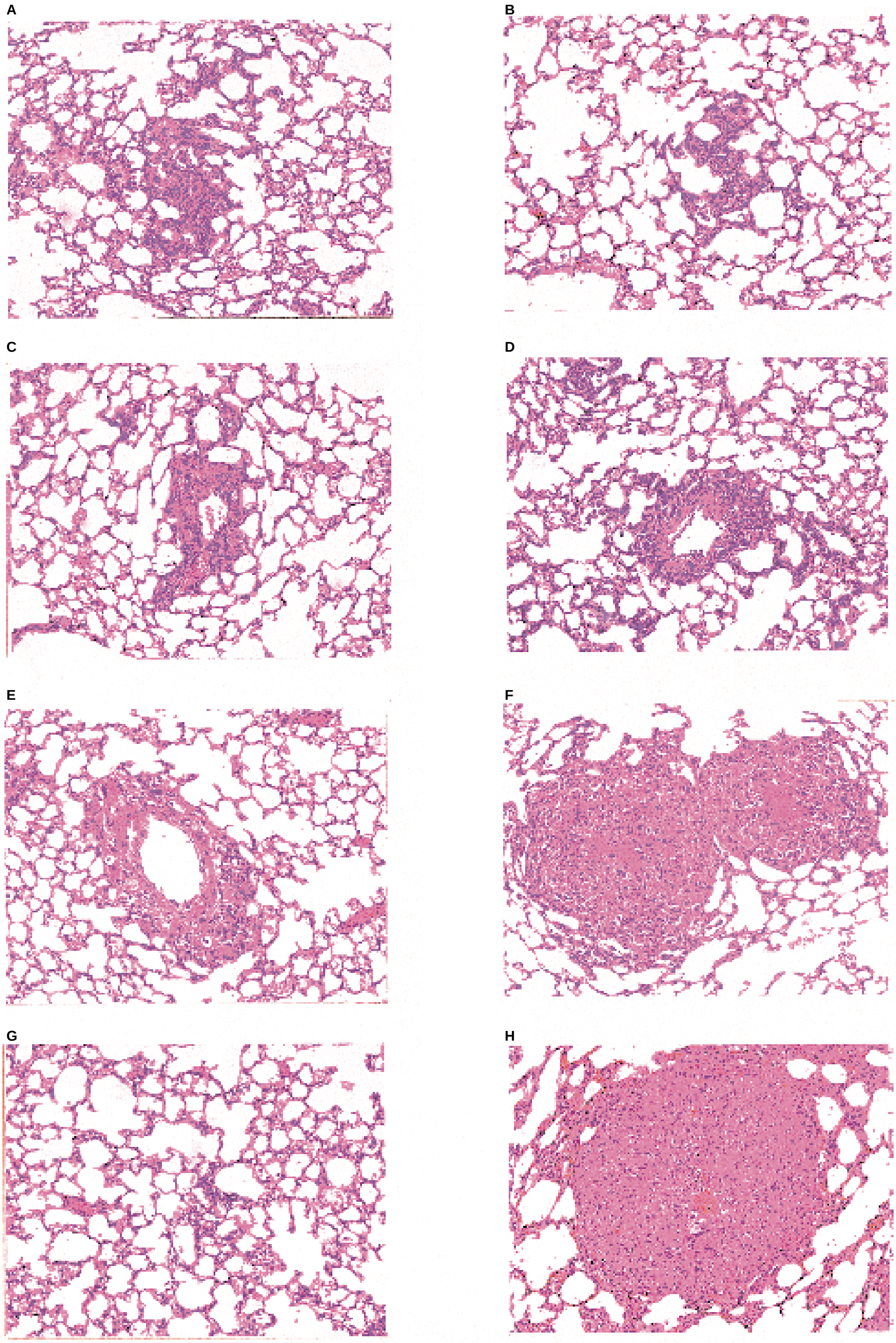

**Figure 1** Time course of EMT-6 tumour treatment in BALB/c lung. BALB/c Bd 8-week-old female mice were injected i.v. with $2 \times 10^4$ EMT-6 cells passage 10. Five days later they were injected i.v. with 24 µg (4.3 MBq) of $^{213}$Bi–mAb 201B (**A**, **C**, **E** and **G**, or with 24 µg mAb 201B with no $^{213}$Bi (**B**, **D**, **F** and **H**). Animals were sacrificed and their lungs inflated for histology at 4 h (**A**, **B**); 1 day (**C**, **D**) 3 days (**E**, **F**) and 7 days (**G**, **H**). Representative tumour sites were video captured and processed using Adobe Photoshop. Approximate magnification 100×

*© Cancer Research Campaign 1999*

chelator molecules for each mAb (Kennel and Mirzadeh, 1997). $^{213}$Bi was eluted from an $^{225}$Ac generator (Boll et al, 1997) in 300 µl of 0.15 M HI, neutralized with 3 M NaAc and reacted with 50–200 µg-derivatized mAb for 6 min, followed by addition of 2 µl of 0.2 M EDTA. The product was diluted in 2-(N-morpholina) ethane sulforic acid (MES) buffer, tested for extent of radiolabelling by use of a Microcon 30 centrifugation filter and injected without purification into animal tail veins in 200 µ1 volumes. Radiolabel incorporation ranged from 80 to 90%.

Isotope distribution studies in animals with tumours were done essentially as described previously (Hughes et al, 1989). Standard samples were counted in an automatic γ-counter (440 KeV, γ) with the test tissue samples. Corrections were made for isotope decay using $t^1/_2$ = 45 min. For dosimetry calculations, kinetic data gathered from animals were fitted to one- or two-compartment exponential functions using the SAAM II software (Foster and Barrett, 1997). The resultant time–activity curves were integrated to obtain organ residence times (Loevinger et al, 1988) for $^{213}$Bi (and daughters). S-values were developed for a 30 g mouse using the MCNP radiation transport software (Briemeister, 1993; Yoriyaz and Stabin, 1997). The mouse model was primarily used to obtain electron self-and cross-doses. α-particle radiation was assumed to be 100% locally absorbed and photon doses were not considered significant, as they represented <1% of the total dose.

### Animal studies

Cells were recovered from frozen storage and passaged once before harvest. Plated cells were harvested when 80% confluent by treatment with trypsin–EDTA. Cells were washed from the plates in growth medium containing FBS to neutralize residual trypsin, counted in a haemocytometer, collected by centrifugation and diluted to the appropriate concentration (see Table legends) in PBS. All cells were injected in 200 µl volumes i.v. in a tail vein using a 27-gauge needle within 30 min of harvest.

At designated times, chelated mAbs were prepared, checked for radiolabelling efficiency, quantitated by monitoring the γ-ray emission in a well-type Na(Tl) gamma detector, diluted to the appropriate concentration without purification and injected i.v. in 200 µl MES. Treated animals were housed in a designated radiation area for 1 day and then transferred to a regulated SPF barrier room. Animals were observed daily and sacrificed when moribund or when external tumours exceeded 1 cm$^3$ in volume.

Necropsy was performed on animals sacrificed by cervical disarticulation. Neutral buffered formalin (0.6 ml) was instilled into the lung through a tracheal catheter and the trachea ligated with standard cotton thread. Organ samples and the heart–lung block were fixed by submersion in buffered formalin for 24 h. Each pulmonary lobe was embedded separately. Lobes were cut along the longitudinal axis of their bronchi and the resulting two halves were embedded face down in paraffin. Sections of 5 µM were cut from blocks trimmed to give maximum tissue cross-section exposure and then stained with haematoxylin and eosin. Lung tumours in a single section per animal were enumerated in a ‘double blind’ fashion using a dissecting microscope.

### RESULTS

### Distribution and dosimetry

We have shown previously that iodinated mAb 201B accumulates in the lung rapidly, resulting in a large fraction of the total dose (up to 50%) accumulating within 5 min post injection (Hughes et al, 1989). For experiments on treatment of lung colonies (artificial metastases), the distribution of $^{213}$Bi–mAb 201B and that of control mAb 14 have been determined as a function of time in mice bearing EMT-6 tumours. Data in Table 1 show that even if the chelation mixture is used without purification, about 33% of the injected dose of $^{213}$Bi is delivered to lung (> 200% ID per g) and this fraction remains essentially constant from 1 to 6 h post injection. In contrast, control mAb 14 delivers about 1% of the dose to lung (< 10% ID perg). $^{213}$Bi that is not attached to mAb is rapidly cleared from the circulation (< 1 h) and eliminated in the urine (Kennel and Mirzadeh, 1997).

Decay of $^{213}$Bi is 98% β-particle (0.41 MeV average) to $^{213}$Po and 2% α (5.8 MeV) to $^{209}$Tl. $^{213}$Po ($t^1/_2$ = 4 µs) decays by α-particle (8.3 MeV) to $^{209}$Pb. $^{209}$Pb was released by the chelator and decays by β-particle (0.214 MeV average) to stable $^{209}$Bi. $^{213}$Bi decay emits a 440 KeV γ (26%). Dosimetry calculations were done to determine the direct dose of α-particle plus β-particle irradiation to the lung and the ‘cross-dose’ of β-particles to the lung from other organs. The model utilized the time activity data of Table 1 and a specific exponential retention function to estimate the residence times of the $^{213}$Bi in each organ. As expected, the absorbed dose of α-particle irradiation delivered to the lung by mAb 201B was more than tenfold higher than for any other organ, and was 30-fold higher than that delivered to the lung by control mAb 14. The absorbed dose of β-particle irradiation delivered to the lung from $^{213}$Bi–mAb 201B was due predominantly to $^{213}$Bi in the lung with little (< 5%) cross-irradiation contributed from the liver or other nearby organs. Overall, the total β-particle dose was 50-fold less than the α-particle dose. The β-particle dose from $^{213}$Bi–mab 14 had a total cross-irradiation component to the lung of about 20%, but the total dose to the lung from this control mAb was less than 5% of that delivered by the specific MAb. Thus, the data show that α-particle irradiation from the specific mAb was by far the dominant factor in absorbed dose to the lung. The microdistribution of α-particle irradiation is also important. Previous work has shown that mAb 201 binds uniformly to the lung endothelium (Kennel et al, 1990) and thus would be expected to deliver the majority of absorbed dose within 60–100 µ of this position. The position of the capillaries in alveolar septa relative to the tumours can be seen in Figure 1. An autoradiographic photomicrograph of the position of mAb 201B relative to the tumour cells has been published (Mori et al, 1995).

### Treatment of EMT-6 tumours

When injected i.v., the majority of EMT-6 cells lodged in the lung, and some formed lung colonies in perivascular areas and in the alveolar parenchyma (Figure 1). The cell dose used resulted in about 100 colonies per lung which, when left untreated, killed the mice within 13–20 days post-injection. A fraction (10–20%) of the animals cured of lung tumours (see below) developed tumours at later times at sites outside the lung – primarily at subcutaneous (s.c.) sites in the extremities. These animals were sacrificed as tumours approached 1 g in size (30–60 days post-injection). The remaining animals, cured of tumours, developed lung disease that was fatal starting at day 80 onward. All animals in the study were examined by gross necropsy and histology to help determine the cause of death.

Several experiments were done to test the therapeutic effect of $^{213}$Bi–mAb 201B on EMT-6 tumours resulting from i.v. injection. Dose–response data in Table 2 show that two of seven animals

© Cancer Research Campaign 1999

**Table 3** Fractionated dose treatment of EMT-6 tumours in BALB/c mice

| | Treatment[a] | | | Lung tumours | | Time to sacrifice[c] |
|---|---|---|---|---|---|---|
| Group | MAb | μg | $^{213}$Bi (MBq) | Animals with tumours/number treated | Tumours per lung[b] | (days ± s.d.) |
| 1 | 201B | 8 | 0 | 7/7 | 99 ± 13 | 13.8 ± 0.7 |
| 2 | 201B | 12 | 2.44 | 0/7 | – | 97 ± 41 |
| 3 | 14 | 12 | 2.29 | 6/7 | 96 ± 103 | 28 ± 8 |
| 4 | 201B | 6/10/11 | 1.0/0.7/1.0 | 6/7 | 13 ± 6 | 39 ± 39 |
| 5 | 14 | 7/10/11 | 1.0/0.7/1.0 | 7/7 | 129 ± 55 | 16 ± 1.3 |
| 6 | 201B | 22 | 4.3 | 1/7 | 150 | 100 ± 39 |
| 7 | 201B | 11/10/11 | 2.4/1.7/1.7 | 6/7 | 7 ± 7 | 50 ± 20 |

[a]BALB/c ♀ mice from Taconic Farms were injected i.v. with $4 \times 10^4$ EMT-6 (passage 12) cells. Animals were treated with $^{213}$Bi–mAb at day 5. Dose fractions were given at 3-day intervals. [b]Significance levels for lung tumour numbers: specific mAb efficacy group 4 vs group 5, $P \sim 0.001$ (Experiment 7 January 1997) [c]Significance levels for time to sacrifice: labelled mAb therapy: groups 2, 6 and 7 all vs group 1; $P < 0.001$; split dose analyses: group 2 vs group 4, $P \sim 0.001$; group 6 vs group 7, $P \sim 0.01$. specific mAb therapy: group 2 vs group 3, $P = 0.018$; group 4 vs group 5, $P \sim 0.01$.

**Table 4** Treatment of EMT-6 tumours in C3H SCID mice

| | Treatment[a] | | | Lung tumours[c] | | Time to sacrifice[d] |
|---|---|---|---|---|---|---|
| Group | mAb | μg | $^{213}$Bi (MBq) | Number positive/tested | Tumours per lung | (days ± s.d.) |
| 1 | 201B | 8 | 3.0 | 5/5 | 7.2 ± 4.3 | 25 ± 4 |
| 2 | 14 | 21 | 2.6 | 5/5 | 88.6 ± 37 | 17.8 ± 1.3 |
| 3 | 201B[b] EDTA | 8 | 3.0 | 4/4 | 111 ± 25 | 15.8 ± 0.5 |
| 4 | 201B | 8 | 2.6 | – | – | 83 ± 14 |

[a]C3H-SCID mice injected i.v. with $2 \times 10^4$ EMT-6 cells (passage 14) except for group 4. Animals were treated 5 days later with i.v. injections of $^{213}$Bi-mAb. [b]$^{213}$Bi mixed with EDTA and mAb to prevent coupling of the mAb with the isotope. [c]Significance levels: group 1 vs group 2; $P \sim 0.001$; group 1 vs group 3, $P < 0.001$. [d]Significance levels group 1 vs group 2, $P = 0.003$; group 1 vs group 3, $P \sim 0.001$ (Experiment 10 April 1997).

**Table 5** Treatment of Line 1 lung carcinoma in BALB/c mice

| | Treatment[a] | | | | | |
|---|---|---|---|---|---|---|
| Group | mAb | μg | $^{213}$Bi (MBq) | Day treated | Animals | Tumours per lung ± s.d.[b] |
| Experiment 1 | | | | | | |
| 1 | 201B | 40 | 6.0 | 6 | 10 | 20 ± 15 |
| 2 | 14 | 40 | 5.4 | 6 | 10 | 35 ± 10 |
| Experiment 25 | | | | | | |
| 3 | 201B | 22 | 4.9 | 5 | 5 | 13 ± 8 |
| 4 | 14 | 22 | 4.9 | 5 | 4 | 55 ± 11 |
| 5 | 201B | 22 | 4.9 | 6 | 5 | 25 ± 10 |
| Experiment 3 | | | | | | |
| 6 | 201B | 13 | 3.7 | 5 | 9 | 11 ± 8 |
| 7 | 14 | 14 | 3.7 | 5 | 10 | 43 ± 15 |
| 8 | 201B(EDTA) | 12 | 3.9 | 5 | 10 | 61 ± 26 |

[a]BALB/c ♀ mice injected i.v. with $1 \times 10^5$ Line 1 cells (passage 12) (Experiment 1, 30 October 1996); $2 \times 10^5$ Line 1 cells (passage 22) (Experiment 2, 14 November 19); $1 \times 10^5$ Line 1 cells (passage 18) (Experiment 3, 10 January 1997). [b]Significance levels for specific mAb to controls. Group 1 vs group 2, $P = 0.025$; group 3 vs group 4, $P < 0.001$; group 6 vs group 7 and group 4 vs group 5,$P = 0.003$; group 6 vs group 8, $P < 0.001$.

treated with as little as 0.925 MBq $^{213}$Bi–mAb 201B were cured of lung tumours. Animals not cured at this dose had fewer lung tumours (12 ± 5 v 78 ± 10 in control animals treated with unlabelled mAb 201B) and died later (33 days ± 12 days) than control animals (14.3 days ± 1.7 days). A dose of 2.6 MBq cured all but one of seven animals and extended lifespan to 98 days ± 56 days. Doses of 3.33 and 6.7 MBq on specific mAb 201B completely eradicated the lung tumour colonies in this experiment. The time to sacrifice in these groups was: 3.33 MBq, 86 days ± 39 days; 6.7 MBq, 50 days ± 26 days. A total dose administered as three

*© Cancer Research Campaign 1999* 

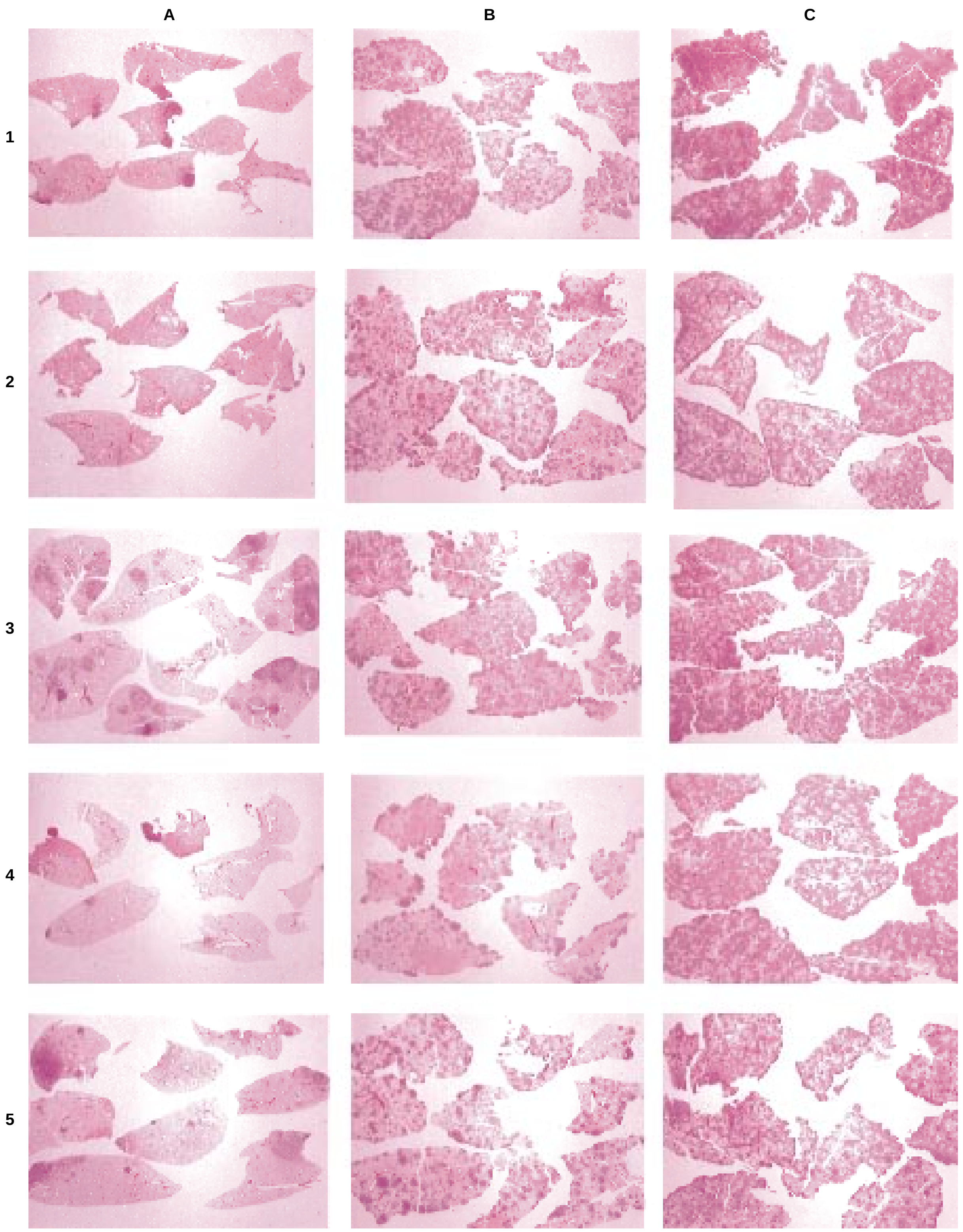

**Figure 2** Photomicrographs of lung sections from treated and control SCID mice which had been injected with IC-12 rat carcinoma. Seven-week-old ICR SCID male mice were injected i.v. with $1 \times 10^6$ IC-12 cells. Individual animals were treated at day 7 with 20 µg (3.0 MBq) of $^{213}$Bi–mAb 201B (**A** 1–5); 20 µg (2.7 MBq) $^{213}$Bi–mAb 14 (**B**) or 20 µg unlabelled mAb 201B (**C**). Animals were sacrificed when moribund (**A** 47 ± 7 days; **B**, 19.4 ± 3 days; **C**, 14.8 ± 0.8 days) and lungs were processed for histology. Approximate magnification = 2×

 *© Cancer Research Campaign 1999*

**Table 6** Treatment of human carcinomas in ICR SCID mice

| | mAb | µg | $^{213}$Bi (MBq) | Day of treatment | Number positive/tested | Tumours per lung[c] |
|---|---|---|---|---|---|---|
| | Treatment[a] of A431 tumours | | | Lung tumours | | |
| 1 | 201B | 13 | 2.8 | 11 | 10/10 | 19 ± 10 |
| 2 | 14 | 13 | 2.8 | 11 | 10/10 | 126 ± 53 |
| 3 | – | – | 0 | – | 4/4 | 127 ± 85 |
| | Treatment[b] of A549 tumours | | | Lung tumours | | |
| 4 | 201B | 13 | 2.8 | 10 | 4/6 | 4.5 ± 6[d] |
| 5 | 14 | 13 | 2.8 | 10 | 5/6 | 55 ± 83 |
| 6 | 201B (EDTA) | 13 | 2.8 | 10 | 5/5 | 86 ± 72 |

[a]ICR SCID mice injected with $7.5 \times 10^5$ A431 cells (passage 34) (Experiment 6 January 1997). [b]ICR SCID mice injected with $10^6$ A549 cells (passage 52) (Experiment 10 January 1997). [c]Group 1 vs group 2, $P < 0.001$ (significant); group 4 vs group 5, NS; group 4 vs group 6, $P = 0.037$. [d]Tumour lesions haemorrhagic but still contain viable tumour cells.

fractions of $^{213}$Bi–mAb 201B did not kill the lung tumours as well as did a single dose.

The histologic appearance of lungs of animals in these treatment groups is shown in Figure 1. Most tumours grew perivascularly (although some grew in the parenchyma) and were therefore good targets for α-particle-mediated therapy. Representative areas of lung sections from animals 5 days after i.v. injection with EMT-6 cells are shown, either treated with radiolabelled mAb 201B (Figure 1 A, C, E, G) or unlabelled MAb 201B controls (Figure 1 B, D, F, H). Each animal had about 100 tumours in the evaluated sections (see Methods for histology sampling) of about 10–50 cells in cross-section. Each tumour contained 150–1500 cells at the time of treatment. At 4 h post-treatment, no differences in tumour morphology were noted (Figure 1 A vs B). At this time the tumour colonies were of only microscopic size, consisting of one or two cell layers. At 24 h, the $^{213}$Bi-treated animals demonstrated cytoplasmic swelling of tumour cells and an apparent arrest in tumour growth (Figure 1C), whereas the control tumours had developed into colonies comprised of several cell layers (Figure 1D). By day 3, the tumour colonies in the animals given $^{213}$Bi were necrotic (Figure 1E), whereas the tumours in mice treated with unlabelled mAb 201B had continued to grow (Figure 1F). Tumours in the control group continued to grow and, by day 7 (Figure 1H), occupied a significant proportion of the lung. This resulted in laboured breathing and the animals were sacrificed. By contrast, the lungs of treated animals were essentially tumour free at this time with scattered focal areas of cellular debris (Figure 1G). Staining for apoptotic cells in these sections, treated as well as untreated animals, remained constant at 2–5% of tumour cells, although cells in spleens of treated animals showed a dramatic, dose-dependent increase in staining for apoptotic cells (data not shown). An unexpected result in this experiment was that $^{213}$Bi control mAb 14 had a significant therapeutic effect especially at high dosage (see Discussion).

As reported earlier (Kennel et al, 1998), animals cured of tumours by treatment with $^{213}$Bi–mAb 201B developed lung disease later in life. Animals treated with 2.6 MBq develop lung disease at about 130 days; 3.33 MBq at 110 days; and 6.7 MBq at 85 days. Fractionated doses of labelled mAb were given to attempt to alleviate this complication. Data in Table 3 (and Table 2) show that fractionated doses of labelled mAb were less effective in tumour therapy than single doses (Table 3, group 2 vs group 4; and group 6 vs group 7). Survival times of specific $^{213}$Bi–mAb 201B-treated animals were all significantly longer than those treated with control $^{213}$Bi–mAb 14 at comparable injected doses (group 2 vs group 3, $P = 0.1$) and (group 4 vs group 5, $P = 0.01$). It should be noted that the time to sacrifice presented here includes death due to lung tumours, s.c. tumours and pulmonary fibrosis. Numbers of tumour colonies per lung, although quite variable in treated animals, were significantly reduced when values for specific mAb groups were compared to those for controls (group 2 vs group 3, $P = 0.018$; group 4 vs group 5, $P = 0.001$).

To determine if immune competence of the animals was necessary for tumour cures, an experiment was done with EMT-6 cells injected into C3H SCID mice. Data in Table 4 show that mice treated with $^{213}$Bi–mAb 201B (group 1) lived significantly longer and had fewer lung tumours than mice treated with $^{213}$Bi–mAb 14 (group 2) or $^{213}$Bi–EDTA mixed with, but not chelated to, mAb 201B (group 3), even though all animals eventually died of lung tumour burden.

### Treatment of Line 1 carcinomas

Similar to the EMT-6 tumour cells, BALB/c Line 1 lung carcinoma cells injected i.v. lodged primarily in the lung. Lung colonies were more nodular and avascular than for EMT-6 tumours and, as such, represented a poorer target for vascular-targeted radioisotope. Line 1 cells are very malignant and do not induce a detectable immune response in the syngeneic host. This cell system is a good model for study of treatment of avascular, non-immunogenic tumours; however, i.v. injection resulted in many extrapulmonary tumours. While only a small fraction of EMT-6-injected animals developed tumours outside of the lung, virtually all of the animals injected i.v. with Line 1 cells developed tumours in the heart muscle or chest wall in addition to their lung tumours. The heart tumours proved fatal early after injection (15–20 days) and they could not be cured in the model system which localized $^{213}$Bi–mAb 201B in the lung. Consequently, all the animals tested in the Line 1 system died of heart/chest tumours. However, at the time of sacrifice, the numbers of tumours in the lung were significantly reduced by treatment with specific $^{213}$Bi–mAb 201B (Table 5). The three experiments reported all showed significant decreases of lung tumours in animals treated with $^{213}$Bi–mAb 201B versus those treated with $^{213}$Bi–mAb 14, even if the treatment was

© Cancer Research Campaign 1999

delayed 1 day (to day 6) when tumours were larger. Treatment at day 5 (smaller tumours) was superior to treatment of larger tumours (day 6). However, none of the treated animals were totally cured of lung tumours.

### Treatment of IC-12 rat tracheal carcinomas

IC-12 cells injected in SCID mice grew perivascularly in a pattern similar to that for EMT-6 cells. ICR SCID mice injected i.v. with IC-12 cells were treated on day 7 with 2.7–3.0 MBq $^{213}$Bi coupled to 20 µg mAb 201B or MAb 14. Low power magnification photomicrographs of the histological sections of lung are shown in Figure 2. The growth pattern of IC-12 cells along blood vessels made it impossible to quantitate actual lung colonies in control animals; however, animals treated with the specific $^{213}$Bi–mAb 201B had a lower tumour burden at sacrifice than did controls. Tumours in treated animals appeared larger, because these animals were sacrificed later than those in the control groups. Animals treated with $^{213}$Bi–mAb 201B (Figure 2A1–5) had a significantly longer lifespan (47 ± 7 days) compared to those treated with $^{213}$Bi–mAb 14 (Figure 2B1–5) (19.3 days ± 3 days) or those treated with unlabelled mAb 201B (Figure 2 C1–5) (14.8 days ± 0.8 days). This result was comparable to that for treatment of EMT-6 tumours in immunodeficient C3H SCID mice (Table 4).

### Treatment of human tumours in SCID mice

Eight human tumour lines were tested for their growth in SCID mice lungs after i.v. injection. Two, A549 and A431, grew as lung colonies at low frequency. Tumour cells of human origin grew much more slowly than rodent tumours. A431 grew as an avascular nodule with the interior cells forming and secreting keratin to the tumour centre as the tumour size increased. Many of the animals developed extrapulmonary tumours and had to be sacrificed. The time to sacrifice was not significantly lengthened by treatment with $^{213}$Bi–mAb 201B. However, histologic examination showed that animals treated with specific $^{213}$Bi–mAb 201B had fewer lung tumours than those treated with control $^{213}$Bi–mAb 14 (Table 6).

A549 was derived from a human lung adenocarcinoma. Intravenous injection of tumour cells resulted in lung colonies in the majority of animals. As for A431, treatment with $^{213}$Bi–mAb 201B did not extend the lifespan of animals, but did result in fewer lung tumours (Table 6). Due to tumour number variability, the differences were marginally statistically significant. Many tumours in animals treated with specific antibody were haemorrhagic (data not shown).

### DISCUSSION

Previous work with α-particle emitters has been done with lymphoid tumours which are accessible to the circulation (Huneke et al, 1992; Hartmann et al, 1994) or with injections of radiolabelled mAb directly to the tumour site (Zalutsky et al, 1994). Since the short-range, high LET α-particle should be useful for local destruction of tumour cells, we devised a model whereby the α-particle emitter was targeted to blood vessels feeding micrometastatic tumours in the lungs.

The present system relies on the targeting of isotopes to normal blood vessels. The specific mAb 201B that reacts with murine TM has been shown to bind efficiently to the lumenal side of endothelial cells lining capillaries and vessels in the lung (Hughes et al, 1989; Kennel et al, 1990). Knowledge of the positioning of the isotope relative to the tumour cells is an advantage in interpreting the results. The radiation dose calculations indicate that most of the lung dose comes from α-particle self-dose, with a much smaller contribution from the lung bound $^{213}$Bi β-particle emission and even less from β-particle cross-dose to the lung from other organs. A more complete description of the dosimetry would be obtained by performing an analysis of the microdosimetry. Humm et al (1987) calculated the absorbed dose to cell nuclei from $^{211}$At positioned in a central capillary. He concluded that, even at very large doses, about 40% of the tumour cells were too far from the source to get a lethal hit. The tumours observed in this study were rather well dispersed throughout the lung, and most were perivascular in origin with cells within about 50 µ from the nearest vessel. Thus, the macrodosimetric estimates shown in Table 1 may be more valid than in some other cases involving therapy with α-particle emitters.

The data show that high doses of $^{213}$Bi–mAb 201B completely eradicated EMT-6 tumours in lungs. High doses of $^{213}$Bi–mAb 14 also had a therapeutic effect in the EMT-6 system at about fivefold higher doses (Tables 2 and 3). $^{213}$Bi complexed with EDTA and mixed with mAb 201B had no significant effect (data not shown). Dosimetry calculations indicate that per input activity mAb 14 should deliver about 30-fold less α-particle dose to the lungs than does mAb 201B. The long circulation times of $^{213}$Bi–mAb 14 relative to that of $^{213}$Bi complexed to EDTA may alter the blood chemistry; however, we have no other plausible explanation for the effect.

The mechanism of tumour eradication is not clear. Photomicrographs in Figure 1 in conjunction with stains for apoptotic cells indicate that EMT-6 cells die between day 1 and 3 and undergo necrosis, but not apoptosis. Debris was cleared over the next week, but areas of the lung where relatively large tumours were present did not regenerate functional lung architecture. This could be the result of killing of the blood vessel cells, depriving the tumour cells of nutrients and oxygen, although other areas of the lung where no tumour was present did not show necrotic cells in this time frame.

The mechanism of antivascular therapy with immunotoxins (Burrows and Thorpe, 1994) or with tissue factor (Huang et al, 1997) was shown to be infarction of tumours due to loss of blood supply. In the current work, targeting was to normal vessels in lung. Since the lung continued functioning for months after treatment, the tumour in the treated lung should have had some blood supply. It is possible that lung vessels are repaired from a repository of endothelial stem cells which avoid damage by the treatment. Thus, temporary or partial damage to the blood vessels may have a more dramatic effect on tumour cells than on the normal lung cells. We have not been able to demonstrate increased vascular permeability in lung vessels serving tumours following treatment with $^{213}$Bi–mAb 201B (data not shown). Given these facts, it is likely that the mechanism of therapy included killing of a significant fraction of tumour cells by α-particles in addition to any affects due to blood vessel damage.

Larger tumours (1–3 mm) were examined early after treatment to determine if the range of cell killing (from the blood vessel) correlated with the α-particle range of 6–10 cell diameters. Cells in larger tumours became necrotic, but it was not clear which cells actually survived and went on to progressive growth, or if the residual live cells originated from areas of the tumour outside of

 *© Cancer Research Campaign 1999*

the α-particle range. We attempted to differentiate dead cells in a ‘kill zone’ around the blood vessel in EMT-6 tumours of various sizes. Neither stains for apoptotic cells nor for tumour cell membrane were adequate to distinguish dead from live cells, up to 7 days after treatment.

Evidence indicated that a functional immune response was necessary to effect cures. EMT-6 is immunogenic in the BALB/c host (Korbelik et al, 1996) and the $^{213}$Bi–mAb 201B-treated EMT-6 lung tumours were completely cured. Treatment of EMT-6 in SCID mice resulted in tumour growth remissions, but not cures, indicating that viable tumour cells remained and grew. Line 1 cells, which are very weakly immunogenic (Yuhas et al, 1975), exhibited residual cells in BALB/c mice which eventually grew back, analogous to the result for the EMT-6 cell system in SCID mice. Similarly, all other tumours, IC-12, A431, and A549, treated in SCID mice regressed, but residual cells ultimately grew to kill the animals. In summary, complete eradication of tumour cells apparently did not occur (at least at doses < 7.4 MBq) and a functional immune system was necessary to deal with residual viable tumour cells.

The rat IC-12 tumour, as well as the human A431 and A549 tumours, represented models of slower growing tumours. The IC-12 tumour grew perivascularly (Figure 2) similar to the pattern for EMT-6 tumours. The lifespan of SCID mice bearing IC-12 tumours was significantly extended by treatment with $^{213}$Bi–mAb 201B, but the animals eventually developed lung tumours. Both human tumours grew more slowly, not developing a lethal tumour burden until about 60 days after i.v. injection. Specific treatment of animals in these models did not result in increased lifespan, but a reduction of tumour colonies was observed in both. The data indicate that even slow growing tumours can be killed by the α-particle irradiation localized in the adjacent vessels as discussed previously.

Unfortunately, treatment with $^{213}$Bi–mAb 201B resulted in damage to normal lung. BALB/c mice treated with 2.6 MBq doses develop lung fibrosis at about 100 days post-treatment. Dose–response data show that animals treated with lower doses developed damage later. A 2.6 MBq dose cured virtually all of the EMT-6 lung tumours, while fibrosis developed at day 130 or later. A dose of 0.925 MBq cured only a few animals of all lung tumours, but fibrosis did not develop in the cured animals observed for up to 1 year. Attempts to minimize lung damage by delivering the dose in fractions, at 3-day intervals, were not as successful in tumour therapy. The 3-day interval should allow for some endothelium (Speidel et al, 1993) and epithelium regeneration (Adamson et al, 1977). The EMT-6 tumours grew so fast that regrowth in the 3-day interval overcame the therapeutic effect of subsequent fractioned doses. For the EMT-6 model, shorter times between treatments are likely necessary to combat tumour regrowth. Fractionated dose therapy of slower growing tumours may be effective with intervals of 3 days or more. These experiments, as well as other approaches to inhibit the fibrotic response, are in progress.

### CONCLUSION

The experimental results described here are for a model system designed to investigate the efficacy and mechanism of vascular targeted α-particle mediated therapy of micrometastases. The data show that significant, specific tumour cell destruction can be accomplished from an α-particle source located in vessels adjacent to the tumour cells. The mechanism of destruction remains unproven but is likely to involve destruction of tumour cells rather than, or in addition to, partial interruption of blood supply. Direct application of lung targeting for treatment of human disease remains problematic. Tumours confined solely to the lung would be the only application, and protection from the collateral normal lung damage would need to be developed. The successful therapy in this murine model systems adds impetus to efforts to identify targets selectively expressed in tumour vasculature (Burrows et al, 1995; Epstein et al, 1995; Pasqualini and Ruoslahti, 1996). Once the correct targeting agents are found, vascular targeting of $^{213}$Bi for therapy of micrometastasis will be an attractive option.

### ACKNOWLEDGEMENTS

We appreciate the kind gift of CHXb-DPTA from Dr Martin Brechbiel in the laboratory of the late Otto Gansow at NIH. Trish Lankford, Linda Foote, Arnold Beets and G-W Chang helped to monitor mice. Jim Wesley provided excellent technical assistance in histology. Ms Beverly Norton helped in the preparation of the manuscript. We also thank Drs M Terzaghi-Howe and RJM Fry for manuscript review and suggestions on the work. Project support was from the ORNL Laboratory Directors Research Development Fund and DOE RFP ERKP038.

### REFERENCES

Adamson IYR, Bowden DH, Cote MG, and Witschi HP (1977) Lung injury induced by butylated hydroxytoluene. *Lab Invest* **36**: 26–32

Blumenthal RD, Sharkey RM, Haywood L, Natale AM, Wong GY, Siegel JA, Kennel SJ and Goldenberg DM (1992) Targeted therapy of athymic mice bearing GW-39 human colonic cancer micrometastases with $^{131}$I-labeled monoclonal antibodies. *Cancer Res* **52**: 6036–6044

Boll RA, Mirzadeh S and Kennel SJ (1997) Optimization of radiolabeling of immunoproteins with $^{213}$Bi. *Radiochimica Acta* **79**: 145–149

Borgström P, Hillan KJ, Sriramarao P and Ferrara N (1996) Complete inhibition of angiogenesis and growth of microtumors by anti-vascular endothelial growth factor neutralizing antibody: novel concepts of angiostatic therapy from intravital videomicroscopy. *Cancer Res* **56**: 4032–4039

Brechbiel MW and Gansow OA (1992) Synthesis of C-functionalized *trans*-cyclohexyldiethylenetriaminepenta-acetic acids for labeling of monoclonal antibodies with the bismuth-212 α particle emitter. *J Chem Soc Perkin Trans I*, **1**: 1173–1178

Briesmeister J (1993) MCNP-A general Monte Carlo n-particle transport code. NCNP User’s Manual. Los Alamos National Laboratory: Los Alamos, CA

Burrows FJ and Thorpe PE (1994) Vascular targeting: a new approach to the therapy of solid tumours. *Pharmacol Ther* **64**: 155–174

Burrows FJ, Derbyshire EJ, Tazzari PL, Amlot,P, Gazdar AF, King SW, Letarte M, Vitetta ES and Thorpe PE (1995) Up-regulation of endoglin on vascular endothelial cells in human solid tumors: Implications for diagnosis and therapy. *Clin Cancer Res* **1**: 1623–1634

Denekamp J (1984) Vasculature as a target for tumor therapy. *Prog. Appl Microcirc* **4**: 28–38

Epstein AL, Khawli LA, Hornick JL and Taylor CR (1995) Identification of a monoclonal antibody, TV-1, directed against the basement membrane of tumor vessels, and its use to enhance the delivery of macromolecules to tumors after conjugation with interleukin 2. *Cancer Res* **55**: 2673–2680

Foster D and Barrett P (1997) Developing and testing integrated multicompartment models to describe a single-input multiple-output study using the SAM II software system. In: *Proc. Sixth International Radiopharmaceutical Dosimetry Symposium*. Oak Ridge Institute for Science and Education: Knokvitte, TN

Giard DJ, Aaronson SA, Todaro GJ, Arnstein P, Kersey JH, Dosik H and Parks WP. (1973) In vitro cultivation of human tumors: establishment of cell lines derived from a series of solid tumors. *J Natl Cancer Inst* **51**: 1417–1423

Hartmann F, Horak EM, Garmestani K, Wu C, Brechbiel MW, Kozak RW, Tso J, Kosteiny SA, Gansow OA, Nelson DL and Waldmann TA (1994) Radioimmunotherapy of nude mice bearing a human interleukin 2 receptor

© Cancer Research Campaign 1999 

alpha-expressing lymphoma utilizing the alpha-emitting radionuclide-conjugated monoclonal antibody 212Bi-anti-Tac. *Cancer Res* **54**: 4362–4370

Huang X, Molema G, King S, Watkins L, Edgington TS and Thorpe PE (1997) Tumor infarction in mice by antibody-directed targeting of tissue factor to tumor vasculature. *Science* **275**: 547–550

Hughes BJ, Kennel S, Lee R and Huang L (1989) Monoclonal antibody targeting of liposomes to mouse lung in vivo. *Cancer Res* **49**: 6214–6220

Humm JL (1987) A microdosimetric model of astatine-211 labeled antibodies for radioimmunotherapy. *Int J Radiat Oncol Biol Phys* **13**: 1767–1773

Huneke RB, Pippin CG, Squire RA, Brechbiel MW, Gansow OA and Strand, M. (1992) Effective α-particle-mediated radioimmunotherapy of murine leukemia. *Cancer Res* **52**: 5818–5820

Kennel SJ, Lankford T, Hughes B and Hotchkiss JA (1988) Quantitation of a murine lung endothelial cell protein, P112, with a double monoclonal antibody assay. *Lab Invest* **59**: 692–701

Kennel SJ, Lee R, Bultman S and Kabalka G (1990) Rat monoclonal antibody distribution in mice: An epitope inside the lung vascular space mediates very efficient localization. *Nucl Med Biol* **17**: 193–200

Kennel SJ and Mirzadeh S (1997) Vascular targeting for radioimmunotherapy with $^{213}$Bi. *Radiochimica Acta*, **79**: 87–91

Kennel SJ and Mirzadeh S (1998) Vascular targeted radioimmunotherapy with $^{213}$Bi: an alpha particle emitter. *Nucl Med Biol* **25**: 241–246

Kim KJ, Li B, Winer J, Armanini M, Gillett N, Phillips HS and Ferrara N (1993) Inhibition of vascular endothelial growth factor-induced angiogenesis suppresses tumor growth in vivo. *Nature* **362**: 841–844

Korbelik M, Krosi G, Krosi J, and Dougherty GJ (1996) The role of host lymphoid populations in the response of mouse EMT6 tumor to photodynamic therapy. *Cancer Res* **56**: 5647–5652

Loevinger R, Budinger T, Watson E (1988) MIRD primer for absorbed dose calculations. The Society of Nuclear Medicine Inc: New York

Mori A, Kennel SJ, Waalkes M v B, Scherphof GL and Huang L (1995) Characterization of organ-specific immunoliposomes for delivery of 3′,5′-*O*-dipalmitoyl-5-fluoro-2′-deoxyuridine in a mouse lung-metastasis model. *Cancer Chemother. Pharmacol* **35**: 447–456

O'Reilly MS, Brem H and Folkman J (1995) Treatment of murine hemangioendotheliomas with the angiogenesis inhibitor AGM-1470. *J Pediatr Surg* **30**: 325–330

O'Reilly MS, Boehm T, Shing Y, Fukai N, Vasios G, Lane WS, Flynn E, Birkhead JR, Olsen BR and Folkman, J (1997) Endostatin: an endogenous inhibitor of angiogenesis and tumor growth. *Cell* **88**: 277–28

Pasqualini R and Ruoslahti E (1996) Organ targeting in vivo using phage display peptide libraries. *Nature* **380**: 364–366

Rockwell SC, Kallman RF and Fajardo, LF (1972) Characteristics of a serially transplanted mouse mammary tumor and its tissue-culture-adapted derivative. *J Natl Cancer Inst* **49**: 735–749

Speidel MT, Holmquist B, Kassis AI, Humm JL, Berman RM, Atcher RW, Hines JJ and Macklis RM (1993). Morphological, biochemical, and molecular changes in endothelial cells after alpha-particle irradiation. *Radiation Res* **136**: 373–381

Terzaghi-Howe M (1987) Inhibition of carcinogen-altered rat tracheal epithelial cell proliferation by normal epithelial cells in vivo. *Carcinogenesis* **8**: 145–150

Yoriyaz H and Stabin M (1997) Electron and photon transport in a model of a 30-g mouse. *J Nucl Med* **38**: 228

Yuhas JM, Tyoa RE, and Wagner E (1975) Specific and nonspecific stimulation of resistance to the growth and metastasis of the line 1 lung carcinoma. *Cancer Res* **35**: 242–244

Zalutsky MR, McLendon RE, Garg PK, Archer GE, Schuster JM and Bigner DD (1994) Radioimmunotherapy of neoplastic meningitis in rats using an α-particle-emitting immunoconjugate. *Cancer Res* **54**: 4719–4725

*© Cancer Research Campaign 1999*

## References

[PDF_00649] Adamson I. Y., Bowden D. H., Cote M. G., Witschi H. (1977). Lung injury induced by butylated hydroxytoluene: cytodynamic and biochemical studies in mice.. Lab Invest.

[PDF_00651] Blumenthal R. D., Sharkey R. M., Haywood L., Natale A. M., Wong G. Y., Siegel J. A., Kennel S. J., Goldenberg D. M. (1992). Targeted therapy of athymic mice bearing GW-39 human colonic cancer micrometastases with 131I-labeled monoclonal antibodies.. Cancer Res.

[PDF_00657] Borgström P., Hillan K. J., Sriramarao P., Ferrara N. (1996). Complete inhibition of angiogenesis and growth of microtumors by anti-vascular endothelial growth factor neutralizing antibody: novel concepts of angiostatic therapy from intravital videomicroscopy.. Cancer Res.

[PDF_00669] Burrows F. J., Derbyshire E. J., Tazzari P. L., Amlot P., Gazdar A. F., King S. W., Letarte M., Vitetta E. S., Thorpe P. E. (1995). Up-regulation of endoglin on vascular endothelial cells in human solid tumors: implications for diagnosis and therapy.. Clin Cancer Res.

[PDF_00667] Burrows F. J., Thorpe P. E. (1994). Vascular targeting--a new approach to the therapy of solid tumors.. Pharmacol Ther.

[PDF_00675] Epstein A. L., Khawli L. A., Hornick J. L., Taylor C. R. (1995). Identification of a monoclonal antibody, TV-1, directed against the basement membrane of tumor vessels, and its use to enhance the delivery of macromolecules to tumors after conjugation with interleukin 2.. Cancer Res.

[PDF_00683] Giard D. J., Aaronson S. A., Todaro G. J., Arnstein P., Kersey J. H., Dosik H., Parks W. P. (1973). In vitro cultivation of human tumors: establishment of cell lines derived from a series of solid tumors.. J Natl Cancer Inst.

[PDF_00693] Huang X., Molema G., King S., Watkins L., Edgington T. S., Thorpe P. E. (1997). Tumor infarction in mice by antibody-directed targeting of tissue factor to tumor vasculature.. Science.

[PDF_00696] Hughes B. J., Kennel S., Lee R., Huang L. (1989). Monoclonal antibody targeting of liposomes to mouse lung in vivo.. Cancer Res.

[PDF_00698] Humm J. L. (1987). A microdosimetric model of astatine-211 labeled antibodies for radioimmunotherapy.. Int J Radiat Oncol Biol Phys.

[PDF_00700] Huneke R. B., Pippin C. G., Squire R. A., Brechbiel M. W., Gansow O. A., Strand M. (1992). Effective alpha-particle-mediated radioimmunotherapy of murine leukemia.. Cancer Res.

[PDF_00703] Kennel S. J., Lankford T., Hughes B., Hotchkiss J. A. (1988). Quantitation of a murine lung endothelial cell protein, P112, with a double monoclonal antibody assay.. Lab Invest.

[PDF_00706] Kennel S. J., Lee R., Bultman S., Kabalka G. (1990). Rat monoclonal antibody distribution in mice: an epitope inside the lung vascular space mediates very efficient localization.. Int J Rad Appl Instrum B.

[PDF_00711] Kennel S. J., Mirzadeh S. (1998). Vascular targeted radioimmunotherapy with 213Bi--an alpha-particle emitter.. Nucl Med Biol.

[PDF_00713] Kim K. J., Li B., Winer J., Armanini M., Gillett N., Phillips H. S., Ferrara N. (1993). Inhibition of vascular endothelial growth factor-induced angiogenesis suppresses tumour growth in vivo.. Nature.

[PDF_00716] Korbelik M., Krosl G., Krosl J., Dougherty G. J. (1996). The role of host lymphoid populations in the response of mouse EMT6 tumor to photodynamic therapy.. Cancer Res.

[PDF_00721] Mori A., Kennel S. J., van Borssum Waalkes M., Scherphof G. L., Huang L. (1995). Characterization of organ-specific immunoliposomes for delivery of 3',5'-O-dipalmitoyl-5-fluoro-2'-deoxyuridine in a mouse lung-metastasis model.. Cancer Chemother Pharmacol.

[PDF_00728] O'Reilly M. S., Boehm T., Shing Y., Fukai N., Vasios G., Lane W. S., Flynn E., Birkhead J. R., Olsen B. R., Folkman J. (1997). Endostatin: an endogenous inhibitor of angiogenesis and tumor growth.. Cell.

[PDF_00725] O'Reilly M. S., Brem H., Folkman J. (1995). Treatment of murine hemangioendotheliomas with the angiogenesis inhibitor AGM-1470.. J Pediatr Surg.

[PDF_00731] Pasqualini R., Ruoslahti E. (1996). Organ targeting in vivo using phage display peptide libraries.. Nature.

[PDF_00733] Rockwell S. C., Kallman R. F., Fajardo L. F. (1972). Characteristics of a serially transplanted mouse mammary tumor and its tissue-culture-adapted derivative.. J Natl Cancer Inst.

[PDF_00736] Speidel M. T., Holmquist B., Kassis A. I., Humm J. L., Berman R. M., Atcher R. W., Hines J. J., Macklis R. M. (1993). Morphological, biochemical, and molecular changes in endothelial cells after alpha-particle irradiation.. Radiat Res.

[PDF_00739] Terzaghi-Howe M. (1987). Inhibition of carcinogen-altered rat tracheal epithelial cell proliferation by normal epithelial cells in vivo.. Carcinogenesis.

[PDF_00743] Yuhas J. M., Toya R. E., Wagner E. (1975). Specific and nonspecific stimulation of resistance to the growth and metastasis of the line 1 lung carcinoma.. Cancer Res.

[PDF_00746] Zalutsky M. R., McLendon R. E., Garg P. K., Archer G. E., Schuster J. M., Bigner D. D. (1994). Radioimmunotherapy of neoplastic meningitis in rats using an alpha-particle-emitting immunoconjugate.. Cancer Res.

